# A Review of Hydroxylated and Methoxylated Brominated Diphenyl Ethers in Marine Environments

**DOI:** 10.3390/toxics10120751

**Published:** 2022-12-02

**Authors:** Ying Zhang, Yi Li, Sijia Li, He Huang, Yezi Chen, Xutao Wang

**Affiliations:** 1 Eco-Environmental Monitoring and Research Center, Pearl River Valley and South China Sea Ecology and Environment Administration, Ministry of Ecology and Environment, Guangzhou 510611, China; 2 School of Marine Sciences, Sun Yat-Sen University, Guangzhou 510006, China; 3 Southern Marine Science and Engineering Guangdong Laboratory (Zhuhai), Zhuhai 519000, China

**Keywords:** OH-PBDEs, MeO-PBDEs, marine environment, bioaccumulation, biomagnification

## Abstract

Hydroxylated polybrominated diphenyl ethers (OH-PBDEs) and methoxylated polybrominated diphenyl ethers (MeO-PBDEs) are present in the marine environment worldwide. Both OH-PBDEs and MeO-PBDEs are known natural products, whereas OH-PBDEs may also be metabolites of PBDEs. There is growing concern regarding OH-PBDEs as these compounds seem to be biological active than PBDEs. In the present study, we reviewed the available data on the contamination of OH/MeO-PBDEs in the marine environment worldwide, including seawater, marine sediment, marine plants, invertebrates, fish, seabirds and mammals. Bioaccumulation and biomagnification of OH/MeO-PBDEs in the marine food web were summarized as well. This study also proposes the future research of OH/MeO-PBDEs, including the production and the synthesis pathway of OH/MeO-PBDEs, the toxicokinetics of OH/MeO-PBDEs and the toxicology and human exposure risk assessment.

## 1. Introduction

Polybrominated diphenyl ethers (PBDEs) were synthesized in the 1860s and have been used as an additive flame retardant in plastics, textiles, electronic circuitry and other materials to prevent fires. Due to their persistence, bioaccumulation, toxicity and long-range transport, PBDEs were listed as the Annex A of Persistent Organic Pollutants under the Stockholm Convention [1]. The environmental pollution caused by PBDEs is a widespread concern.

Along with the deepening of research on PBDEs, hydroxylated and methoxylated polybrominated diphenyl ethers (OH/MeO-PBDEs) have attracted increasing concern in recent years. OH/MeO-PBDEs have been detected in various marine organisms, including biota at a low trophic level (algae, sponges, invertebrates) [2,3,4,5] to those at a high trophic level (seals, whales, polar bears) [6,7,8,9] from all over the world. In addition, OH/MeO-PBDEs have been detected in marine abiotic environmental matrices, such as sea water and sediment [3,10,11,12]. It is noteworthy that OH/MeO-PBDEs were also detected in humans [13,14,15,16,17].

The toxicities of OH/MeO-PBDEs are considered to be comparable to PBDEs because of their similar structures. OH-PBDEs are also structurally similar to thyroid hormones. In vitro exposure experiments have shown that OH-PBDEs exhibit higher neurotoxicity and endocrine-disrupting potency than PBDEs [18,19], as well as greater dioxin-like activity than MeO-PBDEs [20]. Moreover, MeO-PBDEs exert stronger effects on the mRNA abundance of steroidogenic enzymes than OH-PBDEs in the H295R cell line [21].

Based on ^14^C isotope analysis, Guitart et al. (2011) proposed that there are unidentified producers thriving on the surface of the ocean as planktonic organisms and synthesizing MeO-PBDEs and OH-PBDEs [22]. Given the interest in the toxicity of PBDEs and the structural similarities between each of these classes of compounds, there is interest in the sources, distribution and bioaccumulation of OH/MeO-PBDEs in the marine system. This study reviewed the environmental behaviour of OH/MeO-PBDEs in various marine environmental matrices and proposed the future research directions for OH/MeO-PBDEs in marine environments.

## 2. OH/MeO-PBDEs in Seawater

There are few reports on OH/MeO-PBDEs in seawater due to their relatively high log *K*ow values (6.33 and 6.34 for 2′-MeO-BDE 68 and 6-MeO-BDE 47, respectively) [12] and low water solubility. The concentrations of OH/MeO-PBDEs in seawater are shown in Table 1. The mean concentration of ƩOH-PBDEs was 3.0 ± 1.8 pg/L in the seawater from the surrounding areas of the Arctic Yellow River Station, where only 6-OH-BDE 47 and 2′-OH-BDE 68 were detected in seawater, with the concentrations of 0.94–1.8 pg/L and not detected (nd)—4.0 pg/L, respectively [3]. OH-PBDEs were also reported in the southern part of Korea and San Francisco Bay, with a range of concentrations from nd to 18.5 pg/L and nd to 40, respectively [4,10]. MeO-PBDEs were detected in seawater from the southern part of Korea with concentrations of nd—8.18 pg/L but not detected in seawater around the Arctic Yellow River Station [3,4,23]. MeO-PBDEs were also found in seawaters collected from the Great Barrier Reef by means of passive sampling with estimated mean concentrations for 2′-MeO-BDE 68, 6-MeO-BDE 47 and 2′,6-diMeO-BDE 68 of 41, 58 and 4 pg/L, respectively [12].

Higher levels of naturally occurring *ortho*-substituted OH- and MeO-PBDEs were found in seawaters compared to *meta*- and *para*-substituted compounds, suggesting the natural formation of OH- and MeO-PBDEs as a major mechanism in marine environments. *meta*- and *para*-substituted OH- and MeO-PBDEs were suggested to come from interconversion or biotransformation, rather than being formed naturally [24]. This composition, to a certain extent, supports the speculation that there are unidentified producers of these natural OH- and MeO-PBDEs in the surface water of the ocean [22].

## 3. Marine Sediments

OH/MeO-PBDEs were mainly reported in marine sediment around the North Pacific Ocean and the two polar regions (Table 2). The concentrations of OH/MeO-PBDEs in the Antarctic sediment (2850 ng/g dw for OH-PBDEs and 6900 ng/g dw for MeO-PBDEs) were greater than those in the Arctic sediment (median concentrations of 296 pg/g dw and 108 pg/g dw for ƩOH-PBDEs and ƩMeO-PBDEs, respectively) [3]. The compositions of OH-PBDEs and MeO-PBDEs also showed differences between the two polar regions, where 6-MeO-BDE 47 (76%) and 6-OH-BDE 47 (80%) were the most abundant congeners in the Arctic sediments and 6-MeO-BDE 85 (53%) and 6-OH-BDE 85 (37%) were the two main congeners in the Antarctic sediments [3].

The concentrations of OH/MeO-PBDEs appear to be much higher in marine sediments from the west coast of the Pacific Ocean than those from the east coast, and the highest concentrations of ƩOH-PBDEs (1270 pg/g dw) and ƩMeO-PBDEs (59,400 ng/g dw) were found in sediments from southern South Korea [4,23], with *tetra*-MeO- and OH-PBDEs accounting for 30–40%. Higher concentrations of PBDEs were also detected in sediments from South Korea (162–7090 pg/g dw) than those from the west coast of the Pacific Ocean (nd—1600 pg/g dw), suggesting that PBDE levels may be one of the factors to explain the difference between Pacific coasts. However, more research should be carried out to better explore the difference.

In China, OH/MeO-PBDEs were found to be much higher in marine sediments from the East China Sea (11.4−129.1 pg/g dw for ƩOH-PBDEs and nd—838.1 pg/g dw for ƩMeO-PBDEs) and southern Yellow Sea (58.9–402.5 pg/g dw for ƩOH-PBDEs and 43.3–358.4 for ƩMeO-PBDEs) than those in sediments from Liaodong Bay (3.2–116 pg/g dw for ƩOH-PBDEs and 3.8–56 pg/g dw for ƩMeO-PBDEs). Of the 10 OH-PBDEs and 12 MeO-PBDEs, 6-OH-BDE 47, 2′-OH-BDE 68, 6-MeO-BDE 47, 2′-MeO-BDE 68, 6-MeO-BDE 17, 4-MeO-BDE 17, 5-MeO-BDE 47 and 4′-MeO-BDE 101 were detected in sediments from Liaodong Bay with detection frequencies of 100%, 26%, 100%, 87%, 35%, 39%, 4% and 4%, respectively. However, only 6-MeO-BDE 47, 2′-MeO-BDE 68, 6-OH-BDE 47 and 2′-OH-BDE 68 were detected in the sediments from the southern Yellow Sea, with detection frequencies of 89.2%, 40.5%, 100% and 83.8%, respectively. The similar homologues were detected in the sediments from the East China Sea, with detection frequencies of 88.6%, 70.5% and 100% for 6-MeO-BDE 47, 2′-MeO-BDE 68 and 6-OH-BDE 47, respectively.

To explore the source of OH/MeO-PBDEs in marine sediments, Fan et al. (2014 and 2015) investigated the correlations between OH- and MeO-PBDEs and phytoplankton biomarker (brassicasterol, dinosterol and alkenones) concentrations collected from the East China Sea and the southern Yellow Sea, and found significant positive correlations between the concentrations of most compounds [11,25], suggesting that phytoplankton may be the potential producers of OH- and MeO-PBDEs in the marine environment. Although interconversion of 6-MeO-BDE 47 and 6-OH-BDE 47 was investigated in sediment, the much lower conversion ratio from 6-MeO-BDE 47 to 6-OH-BDE 47 (0.010 ± 0.002) [26] and comparable levels of the two compounds in most surface sediments from the East China Sea suggest that a large proportion of 6-OH-BDE 47 may be from the natural production by marine organisms rather than from the conversion from 6-MeO-BDE 47 [25].

**Table 2:** Concentrations of OH/MeO-PBDEs in marine sediments (pg/g dw).

Location	Sample Time	N	ƩOH-PBDEs		ƩMeO-PBDEs		Reference
			Range	Mean/Median	Range	Mean/Median	
The Arctic Yellow River Station	2010–2014	5	137−702	296	7.02–262	108	[3]
The Antarctic Great Wall Station	2010–2014	1		2850 ^a^		6900	[3]
Hudson Bay, Canada	1996–2003	6		<100		<100	[8]
San Francisco Bay, USA	2011	9	<8.1–263.8 ^a^				[10]
Southern coast of South Korea	2015	43	nd—1270	324	6–1560	367	[23]
Southern part of South Korea	2016	16	114–671		5590–59,400		[4]
Liaodong Bay, China	2006	23	3.2–116	24 ± 2.3	3.8−56		[26]
East China Sea	2011	44	11.4–129.1 ^a^		nd—838.1		[25]
Southern Yellow Sea, China	2012	37	58.9–402.5		43.3–358.4		[11]

^a^ Only the concentration of 6-OH-BDE 47 was accessible.

## 4. OH/MeO-PBDEs in Marine Biota

### 4.1. Marine Plants

Concentrations of OH/MeO-PBDEs in marine plants were determined in several locations around the world. The highest concentrations of OH- and MeO-PBDEs (101 and 232 ng/g ww) were found in algae collected from the littoral zones of the Philippines. No obvious difference was observed between concentrations of OH/MeO-PBDEs in algae samples (≤12 ng/g ww) collected from the Baltic Sea, a river estuary in Portugal, the Bohai Sea in China and polar regions (Table 3). Higher levels of OH-PBDEs than MeO-PBDEs were found in red algae collected from both Askö Island and Nämdö Island in the Baltic Sea [27,28] and obviously lower concentrations of PBDEs (1/20 of the MeO-PBDEs) were observed in red algae collected from Askö Island, indicating that both MeO-PBDEs and OH-PBDEs in the red algae are natural products. Moreover, Haraguchi et al. (2010) strongly suggest that OH-PBDEs in algae collected from the littoral zones of the Philippines are of natural origin because their possible precursors such as BDE 47, were undetectable in any of the algae [29].

In red algae collected from Askö Island in the Baltic Sea, 6-OH-BDE 99, 6-OH-BDE 137, 6-OH-BDE 85 and 6-OH-BDE 47 were the four abundant OH-PBDE congeners, with the mean concentrations of 2.5, 2.2, 2.0 and 1.9 ng/g ww, respectively, and MeO-PBDEs were dominated by 6-MeO-BDE 137 (0.36 ng/g ww) and 6-MeO-BDE 47 (0.19 ng/g ww) [28]. A similar profile was found in the same algal species collected from Nämdö Island in the Baltic Sea [27]. However, in macroalgae collected from Portugal and Europe, the levels of *ortho*-substituted congener 2′-MeO-BDE 68 was higher only in springtime and even not detected in Autumn and Winter in comparison to *meta*- and *para*-substituted congeners [2]. *Ortho*-substituted congeners of OH- and MeO-PBDEs were dominated in algae collected from the Bohai Sea of China and the littoral zones of the Philippines in Asia. For instance, only 2′-MeO-BDE 68, 6′-MeO 47, 2′-OH-BDE 68 and 6-OH-BDE 47 were detected in algae collected from the Bohai Sea. Moreover, in algae collected from the Philippines, the concentrations of 2′-MeO-BDE 68 and 6-OH-BDE 47 reached at 229 and 91 ng/g ww, respectively.

**Table 3:** Concentrations of OH/MeO-PBDEs in marine biotas (ng/g).

Location	Sample Time	Species	ƩOH-PBDEs		ƩMeO-PBDEs		Reference
			Range	Mean/Median	Range	Mean/Median	
*Marine Plants*							
Arctic	2010–2014	Seaweed (lw) ^a^		0.6 ± 0.4		0.16 ± 0.16	[3]
Askö Island, Baltic Sea, Europe	2003	Red algae (ww) ^b^	9–12	10	0.29–0.39	0.36	[28]
Nämdö Island, Baltic Sea, Europe	2013	Red algae (ww)	0.16–7.0		0.010–0.15		[27]
Douro river estuary, Portugal, Europe	2019–2020	2 species (ww)			0.08–1.04		[2]
Bohai Sea, China, Asia	2012	5 species (ww)		0.09−5		0.01−0.1	[13]
Littoral zones, Philippines, Asia	2008	3 species (ww)	nd—101		nd—232		[29]
Hudson Bay, Canada, North America	1999–2003	Macroalgae (ww)	nd		nd		[8]
Antarctic	2010–2014	Algae (ww)		1.7 ± 2.0		0.39 ± 0.46	[3]
*Invertebrates*							
Arctic	2010–2014	3 species (lw)		0.9–1.8		0.47–1.5	[3]
Öland Island, Baltic Sea, Europe	2007	Sponges (EOM) ^c^				148	[5]
Vrångskär Island, Baltic Sea, Europe	2008	Blue mussels (lw)		160–3500		160–420	[30]
Baltic Sea, Europe	2011–2012	Blue mussels (lw)	17–1500		27–220		[31]
Nämdö Island, Baltic Sea, Europe	2013	Amphipod crustaceans (ww)	0.62−10		0.37−1.56		[27]
Douro river estuary, Portugal, Europe	2019–2020	2 species (ww)				1.34–5.66	[2]
Southern coast of South Korea, Asia	2015	Bivalves (lw)	nd−30.6		4.68−939		[23]
Tianjin, Weihai and Penglai, China, Asia	2007, 2009–2011	3 species (lw)		5.81 ± 5.43		3.63 ± 3.53	[32]
Liaodong Bay, China, Asia	2006	5 species (lw)		1.7 ± 0.002		2.44–15.96	[26,33]
Southern part of South Korea, Asia	2016	6 species (lw)	2.48–40.7		0.182–4.65		[4]
Coastal area of Dalian, Bohai Sea, Asia	2012	10 species (lw)		nd−63		nd−21	[13]
Hudson Bay, Canada, North America	1996–2003	Bivalves (lw)		nd	3.4–54	14	[8]
Mediterranean coastal, Tunisia, Africa	2015	Urchin (lw)			nd—364	26.7–99.3	[34]
Nikko Bay, Koror, Palau, Oceania	2005–2009	Sponge (EOM)	50,000–119,000		36,500–63,500		[35]
Sydney Harbour, Oceania	2006	2 species (lw)				2.3, 10.8	[36]
Queensland, Australia, Oceania	2008–2017	Green turtles (lw)			nd—267.3		[37]
Antarctic	2010–2014	2 species (lw)		4.6–15		0.37–0.83	[3]
*Fish*							
*Arctic*	2010–2014	Atlantic cod (lw)		0.25 ± 0.09	1.1–2.6	1.8 ± 0.7	[3]
Svalbard, Storfjorden, Europe	2001	Polar Cod (lw)				0.72	[38]
Adriatic Sea, Mediterranean Sea, Europe	2006	2 deep-sea species (lw)			4.8–39	28.9, 6.5	[39]
Stockholm archipelago, Sweden, Europe	2013	2 species (ww)	nd−18		nd—8.3		[27]
Douro river estuary, Portugal, Europe	2019–2020	3 species (ww)			nd—0.51		[2]
Mediterranean Sea, Europe	2003	Bluefin tuna (lw)			47–503		[40]
Southern North Sea, Europe	2008	7 species (lw)			nd—157		[41]
North Pacific Ocean, Asia	1999	Pacific tuna (ww)	0.02–0.04		0.27–0.76		[6]
Coastal area of Dalian, Bohai Sea, Asia	2012	5 species (lw)	nd—0.5		2−17		[13]
Liaodong Bay, Northern China, Asia	2006	8 species (lw)		0.28 ± 0.001		126.3 ± 189.3	[26,33]
Southern part of South Korea, Asia	2016	14 species (lw)	nd−8.76		0.956–61.5		[4]
Hudson Bay, Canada, America	1996–2003	nd	nd		0.7–150	3.0–42	[8]
Bizerte Lagoon, Tunisia, Africa	2009	2 species (lw)			6.46–798		[42]
Mediterranean Sea, Tunisia, Africa	2009	2 species (lw)			190–578		[42]
Nikko Bay, Koror, Palau, Oceania	2005–2009	Unicorn fish (lw)	nd	nd	65–670	280	[35]
Guam Island, Micronesia, Oceania	2005–2009	Surgeon fish (lw)	nd	nd	47–580	290	[35]
Okinawa Island, Japan, Asia	2005–2009	Grouper (lw)	<0.2–24	10	150–2200	550	[35]
Sydney Harbour, Oceania	2006	6 species (lw)			0.7–110.1		[36]
Antarctica	2010–2014	Patagonian toothfish (lw)		0.35 ± 0.30	nd—5.8	2.7 ± 1.7	[3]
*Seabirds*							
Baltic Sea, Europe	1998	Guillemots (lw)				2.04	[43]
Baltic Sea, Europe	2000, 2009	Long-tailed ducks (lw)	3.4–−8.0	6.1	2.3–6.9	3.8	[27]
Baltic Sea, Europe	1992–2004	White-tailed sea eagle (lw)				240–340	[44]
Svalbard, Norwegian Arctic, Europe	2004	Glaucous gulls (ww)	nd—1.05		0.30−4.30		[45]
Norwegian Arctic, Europe	2006	Glaucous gulls (ww)	0.01–22.3		0.05−5.65		[46]
Swedish Baltic Coast, Europe	1996–1998	White-tailed sea eagle (ww)			0.36–1.3	0.44–0.85	[47]
Liaodong Bay, China, Asia	2006	2 species (lw)		0.084 ± 0.001		2.94 ± 2.70	[26,33]
Indian and South Atlantic Oceans, Asia	1992–2002	Albatross (ww)	0.11−0.90	0.34 ± 0.24	0.08−2.8	0.65 ± 0.85	[48]
West Greenland, North America	2009	White-tailed sea eagle (ww)				0.01–3.9; 0.04–6.4 ^d^	[49]
Hudson Bay, North America	1996–2003	2 species (lw)	nd	nd	0.3−13	1.3, 2.1	[8]
South Atlantic Ocean	1995–1996	Albatross (ww)	0.23–0.67		0.16–4.5		[6]
Indian Ocean	1992–1996	Albatross (ww)	0.17−1.4		0.13–3.9		[6]
*Mammals*							
Arctic Ocean	1993–2002	Polar bear (ww)	nd—0.033		0.006–0.056		[6]
Svalbard, Norwegian Arctic, Europe	2002	Polar Bear (ww)	nd—0.54		nd−0.17		[45]
Svalbard, Norway, Europe	2007	Ringed seal (ww)	<0.02–0.11	0.019			[7]
Baltic Sea, Europe	2002, 2006, 2007	Ringed seal (ww)	0.041–1.06	0.36			[7]
Mediterranean Sea, Europe	1990–1992	5 species (lw)			<3–808		[50]
North Sea coasts, Netherlands, Europe	1998–2008	Harbour porpoise (lw)			5–405		[51]
Black Sea, Europe	1998	Harbour porpoise (lw)			0.6–122		[52]
Sub-Arctic and Arctic	1986–2009	7 species (lw)			0.3–653; 0.2–18 ^e^		[53]
Purchased retail in Japan, Asia	1999	4 species (lw)			48.3–2959		[9]
Ishigaki Island, Japan, Asia	2007	2 species (lw)	0.21–14.6		65–843		[54]
Hokkaido, Japan, Asia	2010–2014	14 species (ww)	nd—80		8.6–211		[55]
Japanese coast, Asia	2005–2013	3 species (ww)	0.02–5.2		0.055–8.6		[56]
Ishigaki Island, Japan, Asia	2007	4 species (lw)			60–2700		[57]
Hudson Bay, North America	1996–2003	2 species (lw)	nd—1		1.7–1800		[8]
Atlantic coast, North America	2001–2006	Harbour Seal (ww)	0.07−1.85		0.01–4.40		[58]
East Greenland, North America	2002	Ringed seal (lw)		0.7 ± 0.5		4.6 ± 0.4	[59]
Canada, North America	2002–2003	Beluga Whale (lw)	nd		20–100		[60]
Reunion Island, Indian Ocean, Africa	2010–2011	Humpback whales			nd—2.5		[61]
Zanzibar, Tanzania, Africa	2000–2002	2 species (lw)			600–210,000		[62]
Southeast Brazil, South America	1994–2006	10 species(lw)			33–249,000		[63]
Brazilian States, South America	1994–2009	6 species (lw)			41.1–13,884		[64]
Northeastern Australia, Oceania	1996–1999	2 species (lw)			240–11,000		[65]
South Australia, Oceania	1989–2014	Bottlenose Dolphin (lw)				470–27,000	[66]
Australia coast, Oceania	2008	Long-finned pilot whale (lw)			59–1635		[67]

^a^ The concentrations are presented in ng/g lipid weight. ^b^ The concentrations are presented in ng/g wet weight. ^c^ The concentrations are presented in ng/g on EOM (extractable organic matter). ^d^ The level of ƩMeO-PBDEs was not given, so the mean level ranges of 2′-MeO-BDE 68 (0.01–3.9 ng/g ww) and 6-MeO-BDE 47 (0.04–6.4 ng/g ww) are present here. ^e^ The level of ƩMeO-PBDEs was not given, so the concentration ranges for 2′-MeO-BDE 68 (0.2–18 ng/g ww) and 6-MeO-BDE 47 (0.3–653 ng/g ww) are present here.

### 4.2. Invertebrates

Concentrations of OH/MeO-PBDEs in invertebrates, including sponges, molluscs, coelenterate, crustaceans, urchin and so on, were determined in polar regions and five continents (Europe, Asia, America, Africa and Oceania) (Table 3). The highest concentrations of OH-PBDEs and MeO-PBDEs were found in sponges collected from Nikko Bay, Koror, Palau, in Oceania, with concentration ranges of 50,000–119,000 ng/g extractable organic matter (EOM) and 36,500–63,500 ng/g EOM, respectively, which were about 2–3 orders of magnitude higher than those in invertebrates from other regions. Arctic and Antarctic marine ecosystems are important components of global biodiversity, the levels of ƩOH-PBDEs (0.6–15 ng/g lw) and ƩMeO-PBDEs (0.16–0.83 ng/g lw) in invertebrates collected from the polar regions were comparable to those in the Hudson Bay, Douro river estuary, Portugal and Tianjin, Weihai and Penglai in China but much lower than those in the Baltic Sea and the Southern part of South Korea (Table 3). Levels of OH- or MeO-PBDEs were found to be higher than their parent compounds (PBDEs) in invertebrates from the Antarctic, Douro river estuary in Portugal, Bohai Sea in China and Hudson Bay in Canada [2,3,8,13]. Significantly, correlation was found between the concentrations of Ʃ_7_OH-PBDEs and Ʃ_7_MeO-PBDEs in Baltic mussels with no obvious correlation between Ʃ_7_MeO-PBDEs and ƩPBDEs [31]. These results indicated that OH/MeO-PBDEs were natural products and different from the anthropogenic origin of PBDEs. Moreover, Chool et al. (2018) investigated the possibility of natural formation and source effects with the comparison between chemical levels in the marine environment from the near- and offshore of South Korea. They observed significant differences in the PBDE concentrations between the nearshore and offshore sites (Mann–Whitney *U*-test, *p* < 0.05), with much higher PBDE concentrations in the nearshore whereas all of the study sites had similar levels of OH- and MeO-PBDEs regardless of the distance from land [23], providing evidence of the natural formation of OH/MeO-PBDEs in the marine environment. Löfstrand et al. (2011) investigated the seasonal variations of OH- and MeO-PBDEs in blue mussels from the Baltic Sea and found that both OH-PBDE and MeO-PBDE levels in the mussels had seasonal variations from May to October, with the highest concentration in June. The seasonal variations of OH-PBDEs and MeO-PBDEs strongly indicated that these substances are formed by natural processes, and the timing of the concentration peaks suggests that filamentous macro-alga may be an important source of these compounds in blue mussels in the Baltic Sea [30]. MeO-PBDEs were detected in the muscle, fat, liver and kidneys of green turtles from Queensland in Australia, with concentrations of nd−267.3, 3–199.1, nd—90.4 and nd—41.2 ng/g lw, respectively [37].

*Ortho*-substituted congeners of OH- and MeO-PBDEs were dominant in almost all the invertebrates within the global marine environment. For example, 6-MeO-BDE-47 (82 ± 10%) and 6-OH-BDE-47 (76 ± 15%) were the most abundant MeO-PBDE and OH-PBDE congeners in organisms in both polar regions, followed by 2′-MeO-BDE-68 (14 ± 11%) and 2′-OH-BDE-68 (14 ± 5%) [3]. 6-MeO-BDE 47 (55 ± 12 ng/g EOM) and 2′-MeO-BDE 68 (93 ± 11 ng/g EOM) were the most abundant congeners are in sponges from the Baltic Sea [5]. The predominance (≥75%) of 6-OH-BDE 47, 6-OH-BDE 85 and 6-OH-BDE 90 for OH-PBDEs and 6-MeO-BDE 47, 2′-MeO-BDE 68 and 6-MeO-BDE 99 for MeO-PBDEs were observed in Baltic blue mussels. Moreover, only 2′-MeO-BDE 68 and 6-MeO-BDE 47 were detected in urchins from the Mediterranean coastal area in Tunisia. However, *ortho*-substituted congeners 2′-MeO-BDE 68 and 6-MeO-BDE 47 in mussels from the Douro river estuary in Portugal were statistically higher only in winter in comparison to *meta-* and *para*-substituted congeners. In addition, the higher concentrations of *meta*-substituted MeO-PBDEs at the nearshore than at the offshore were observed in bivalves from South Korea, indicating the influence of inland sources; it can be supported that highest concentrations of the *meta*-substituted MeO-PBDEs were observed among all substitution positions in river soil [68].

The ratio between 2′-MeO-BDE 68 and 6-MeO-BDE 47 was found to be different for estuarine, continental shelf and oceanic organisms [63,64]. Ratios > 1 were found for estuarine and continental shelf species, whereas ratios < 1 corresponded to oceanic organisms. 3.44). Vetter (2006) raised the hypothesis that higher concentrations of 2′-MeO-BDE 68 are caused by sponges or associated organisms, whereas higher proportions of 6-MeO-BDE 47 are an indication of the presence of algae or associated organisms [69]. In the present studies, ratios > 1 have been found in species from estuarine and continental shelf areas, such as urchins from the Tunisian coast and green turtles from Queensland (except for kidney tissue) [34,37].

### 4.3. Fish

The levels of OH-PBDE and MeO-PBDE in marine fishes are provided in Table 3. Compared with the invertebrates, fewer studies have reported OH-PBDE concentrations in fish and concentrations of OH-PBDEs in fishes were generally 1–2 orders of magnitude lower than those in invertebrates from the same region. The much more bioaccumulative nature of MeO-PBDEs in fish than OH-PBDEs (at least regarding 6-OH/MeO-BDE 47 in zebrafish) [70] and the lower half-lives of OH-PBDEs in fish (10–20 days for 2′-OH-BDE 68) [71] may account for these phenomena.

The highest concentration of ƩMeO-PBDEs were detected in groupers (2200 ng/g lw) collected from Okinawa Island in Japan, which seem to be one order of magnitude higher than those in fishes from the Mediterranean Sea (47–578 ng/g lw), southern North Sea (nd—157 ng/g lw), Hudson Bay (0.7–150 ng/g lw), Bizerte Lagoon (6.46−798 ng/g lw), Nikko Bay (65–670 ng/g lw), Guam Island (47–580 ng/g lw) and Sydney Harbour (0.7–110.1 ng/g lw) and 2–3 orders of magnitude higher than those in fishes from the other regions. Liu et al. (2018) observed that large-size greenling contained less OH-PBDEs and MeO-PBDEs than the small-size, which might be caused by their different diet habits [13]. Young greenlings mainly feed on shrimp, cephalopods and polychaetes, whereas the adult greenlings live on fish and shrimp [72]. In fish samples collected from South Korea, ƩMeO-PBDEs were statistically higher in pelagic fish (6.15–61.5 ng/g lw) than in demersal fish (0.956–8.52 ng/g lw) and benthic invertebrates (0.182–4.65 ng/g lw), implying that it may affected by habitat or the surrounding environment [4]. The formation of MeO-PBDEs seemed to be very active in pelagic fish living near the water surface where marine sponges or algae live under a high sunlight intensity because, as reported, phytoplankton communities are a potential source of and an important contributor to the MeO-PBDEs distribution in biota [23,73]. Ameur et al. (2011) investigated the difference in PBDE and MeO-PBDE levels in two fish species collected from the Bizerte Lagoon and the Mediterranean Sea 8 km northwards from the lagoon [42] and found that the ƩMeO-PBDEs concentrations in the two fish species were higher than the ƩPBDEs in both areas, which may indicate the natural origin of the MeO-PBDEs. Moreover, concentrations of MeO-PBDEs in the two species were significantly higher in the Mediterranean Sea than in Bizerte Lagoon (*p* = 0.02). The existence of a difference in MeO-PBDE levels between the two investigated locations is in accordance with the result that concentration levels of MeO-PBDEs are higher in samples collected from oceanic waters than those collected from coastal waters or river waters [36,63].

Similarly to the invertebrates, 6-OH/MeO-BDE 47 and 2′-OH/MeO-BDE 68 were the four most frequently congeners detected in fish, i.e., 6-MeO-BDE 47 and 2′-MeO-BDE 68 were the predominant MeO-PBDE congeners (≥70%) in five fish species from Dalian in China and only 2′-OH-BDE 68 and 6-OH-BDE 47 for OH-PBDEs were detected in the same fish species [13]. The only detectable congeners of 2′-MeO-BDE 68 and 6-MeO-BDE 47 were observed in fishes from Sydney Harbour and the Mediterranean Sea [36,39]. However, *meta*- and *para*-substituted MeO-PBDEs were statistically higher in fish collected from the Douro river estuary in spring and summer than in other seasons; similar results are reported in freshwater fish from South Korea, with predominance of the 5-MeO-BDE 47, 5-MeO-BDE 100 and 4-MeO-BDE 49 congeners. Those findings were associated the possible transformation of MeO-PBDEs into fish species [2,74].

A shift in the ratio of 2′-MeO-BDE 68 to 6-MeO-BDE 47 was observed between wild and farmed tuna collected from the Mediterranean Sea. In the first case, the ratio was close to 2:3, whereas in farmed tuna the ratio dropped to 1:3, indicating that a higher proportion of 2′-MeO-BDE 68 was present in farmed tuna [40]. As suggested by Vetter (2006), a higher contribution of 2′-MeO-BDE 68 would indicate sponges as the dominant source of MeO-PBDEs, whereas a higher proportion of 6-MeO-BDE 47 would point to algae as the principal source of MeO-PBDEs. The fact of a higher level of 2′-MeO-BDE 68 in farmed tuna support this hypothesis and this is explained by the restriction in mobility of farmed tuna and, as a consequence, a reduced direct contact with sponges [69].

### 4.4. Seabirds

Concentrations of OH/MeO-PBDEs in seabirds were reported in various specimens, such as blood, muscle, liver, kidney and eggs (Table 3). In general, it seems no significant difference exists between the concentration levels of OH-PBDEs and MeO-PBDEs in the same seabird samples worldwide, except those from Hudson Bay where no OH-PBDE was detected. At the same sampling site, concentrations of ƩMeO-PBDEs in seabirds were generally lower than those in invertebrates and fish, whereas OH-PBDE levels were comparable with those in fish but universally lower than those in invertebrates, with an exemption of the level of MeO-PBDEs in white-tailed sea eagle eggs from the Baltic Sea (Table 3). The lesser concentrations of both OH-PBDEs and MeO-PBDEs in birds is possibly due to greater biotransformation of the two compounds that have been observed in microsomes of chickens compared with those of trout [6].

The correlations between PBDEs and their hydroxylated/methoxylated analogues were different among seabirds from different regions. In long-tailed ducks from the Baltic Sea, no correlation between ƩOH/MeO-PBDEs and ƩPBDEs were found, furthermore, the concentrations of 6-MeO-BDE 47 and BDE 47 were not correlated in the long-tailed ducks [31]. However, the concentrations of BDE 47 in glaucous gulls from the Norwegian Arctic correlated positively with those of 6-MeO-BDE 47 and 6-OH-BDE 47 and the 6-MeO-BDE 47 and 6-OH-BDE 47 concentrations also correlated with other PBDE congeners analyzed [45]. Strong correlation between PBDEs and MeO-PBDEs was also found in the tissues of white-tailed eagles and the correlations of 6-MeO-BDE 47 and 2′-MeO-BDE 68 between the different PBDE congeners were found very strong and significant [49]. The difference in correlations could be explained by the different diets, metabolisms and other unknown reasons, further research should be performed to identify the potential sources of OH- and MeO-PBDEs in seabirds.

Although the congener profile of OH-PBDEs and MeO-PBDEs in seabirds varies among different regions, *ortho*-substituted MeO-PBDEs were recognized as the most commonly detected congeners in seabirds worldwide. The long-tailed duck from the Baltic Sea was found to have predominant 6-OH-BDE 47 (67% of ƩOH-PBDEs) and 6-MeO-BDE 47 (42% of ƩMeO-PBDEs); however, their main food, mussels, was found to contain several OH-PBDEs and MeO-PBDEs. The difference in congener profile between long-tailed ducks and their prey might be due to metabolic processes, e.g., in vivo debromination and/or selective elimination in the ducks, which was suggested by Dahlberg et al. (2016) [31]. Different MeO-PBDE profiles were also found between aquatic organisms and seabirds from Liaodong Bay, with 6-MeO-BDE 47 the dominant among the eight target MeO-PBDEs in all invertebrate and fish samples and accounting for 76.2 ± 27.2% of the total MeO-PBDEs. In seabirds, the contribution of 6-MeO-BDE 47 relative to the total MeO-PBDE concentration decreased to 20.2 ± 27.8%, whereas 2′-MeO-BDE 68 became the dominant congener (56.2 ± 38.4%) [33]. The different profiles of OH/MeO-PBDEs in seabirds and aquatic organisms have been attributed to congener-specific dietary exposure or differences in biotransformation capacities between avian species [6,31].

### 4.5. Marine Mammals

In marine mammals, the levels of OH-PBDEs were 1–3 orders of magnitude lower than MeO-PBDEs (Table 3); the highest level of ƩOH-PBDEs (80 ng/g ww) was found in liver of Cuvier’s beaked whale from the coast of Hokkaido, Japan [55]. There are two sources of OH-PBDEs in marine mammals, one is from the metabolism of PBDEs or MeO-PBDEs and the other is the accumulation of a natural source. Wan et al. (2009) explored the source of OH-PBDEs in polar bears by investigating the correlation among PBDEs, OH-PBDEs and MeO-PBDEs and found no significant relationship between concentrations of ƩPBDEs and ƩOH-PBDEs; however, significant correlations were found between the concentrations ƩMeO-PBDEs and ƩOH-PBDEs. Significant correlations were also found between concentrations of 6-OH-BDE 47 and 6-MeO-BDE 47. In addition, the variation in patterns among species was similar for OH-PBDEs and MeO-PBDEs. Significant correlations for OH-PBDEs and MeO-PBDEs suggest transformation of the two compounds [6,75]. Moreover, the demethoxylation of MeO-PBDEs to OH-PBDEs was demonstrated by conducting in vivo metabolism of PBDEs, OH-PBDEs and MeO-PBDEs by rainbow trout, chick, and rat. In the exposure experiment, significant amounts of 6-OH-BDE-47 were generated from 6-MeO-BDE-47 and more OH-PBDE congeners were detected when additional MeO-PBDE congeners were incubated with microsomes, even at lesser concentrations (100 ppb) [6]. Except for the metabolic pathway from MeO-PBDEs, McKinney et al. (2006) have shown that biotransformation of PBDEs occurs in vitro in beluga whales; they also found low, but measurable, concentrations of OH-PBDEs in the liver of beluga whales [76]. Several OH-PBDE congeners were also identified in rats after exposure to BDE 47 [77,78]. Moreover, the significant correlations of OH-PBDEs and PBDEs in harbour seals from the northwest Atlantic, together with the predominance of *meta*- and *para*-OH-substituted PBDEs in polar bears from Svalbard in the Norwegian Arctic also supported the metabolic source of OH-PBDEs [45,58]. However, of 14 OH-PBDEs monitored, only 6-OH-BDE 47 were detected in the ringed seal from East Greenland and *ortho*-OH-substituted PBDEs occupied up to 71% in ringed seal from the Baltic sea, suggesting the OH-PBDEs in marine mammals may also be bioaccumulated via natural sources because *ortho*-OH-substituted PBDEs have been detected, for example, in algae, cyanobacteria and marine sponges [7,59].

MeO-PBDEs were universally detected in marine mammals (Table 3); the highest concentration (249,000 ng/g lw) was found in cetaceans from Rio de Janeiro state in Southeast Brazil [63]. Dorneles et al. (2010) have speculated that the benthic transportation of MeO-PBDEs may contribute to the high concentration found in cetaceans from Rio de Janeiro state. The “Região dos Lagos” area is an area strongly influenced by the upwelling phenomenon, which may be contributing to the transport of MeO-PBDEs from the benthic to the pelagic food chain. Squids constitute important prey for the analyzed cetaceans and “Região dos Lagos” is an area where these molluscs are particularly abundant [63]. Moreover, significantly higher concentration levels (600–210,000 ng/g lw) of MeO-PBDEs were also observed in dolphins from Zanzibar, Tanzania, which is also influenced by the upwelling phenomenon [79]. In addition, higher water temperature is also used to explain the higher MeO-PBDEs in marine mammals, as higher water temperatures could be responsible for an increased activity of MeO-PBDE-producing algae or sponges [66].

MeO-PBDEs in marine mammals were generally considered to come from natural sources, since *ortho*-MeO-substituted PBDEs were the predominant or even the only detected congeners for MeO-PBDEs in marine mammals. For example, only 6-MeO-BDE 47 was detected in humpback whales breeding in the Indian Ocean [61] and only 6-MeO-BDE 47, 2′-MeO-BDE 68 and 6-MeO-BDE 85 of 15 MeO-PBDEs were detected in ringed seals from East Greenland [59]. The predominance of 6-MeO-BDE 47 and 2′-MeO-BDE 68 was also observed in dolphins, whales and harbour seals from other areas in the world [62,64,66,67].

In a study of 53 dolphins collected from six locations along the Brazilian coast, different 2′-MeO-BDE 68/6-MeO-BDE 47 ratios were found between the estuarine dolphin species (1.06 ± 0.41) as well as Atlantic-spotted dolphins (1.86 ± 0.75) and the oceanic Fraser’s dolphin (0.34 ± 0.26) [63,64]. Based on the hypothesis raised by Vetter (2006), the authors pointed out that these ratios would imply that both estuarine and CS dolphins would be receiving MeO-BDEs predominantly from sponges or associated organisms, whereas the oceanic species would be receiving MeO-BDEs predominantly from algae or associated organisms.

## 5. Bioaccumulation and Biomagnification of OH/MeO-PBDEs in Marine Biota

### 5.1. BCF/BAF and BSAF

The bioconcentration/bioaccumulation factor (BCF/BAF) and biota–sediment accumulation factor (BSAF) are used frequently to evaluate the toxicity of pollutants in aquatic organisms and to develop environmental standards and guidelines. BCF/BAF > 5000 (log BCF/BAF > 3.7) indicates that aquatic biotas can enrich pollutants from water and BSAF > 1 indicates that pollutants in sediments can be enriched by organisms. The observed median BCF and BSAF values were relatively high for MeO-PBDEs (7.15 × 10^6^ L/kg, 7.06) and OH-PBDEs (1.05 × 10^6^ L/kg, 0.277) in marine biotas from the southern part of South Korea [4], and comparable log_10_BAF values (5.3–6.2) of major congeners of OH-PBDEs were also investigated in marine species from the polar regions [3], suggesting that OH/MeO-PBDEs can be bioaccumulated or formed by diverse mechanisms in biota despite low concentrations in the surrounding environments.

Higher BSAF values for 6-MeO-BDE 47 (0.48–7.2 g TOC/g lipid) and 2′-MeO-BDE 68 (0.14–2.1 g TOC/g lipid) than 6-OH-BDE 47 (0.017–0.96 g TOC/g lipid) and 2′-OH-BDE 68 (0.19–1.5 g TOC/g lipid) were found in the invertebrates from Liaodong Bay [26]. Higher BSAF values for MeO-PBDEs (7.6) than OH-PBDEs (0.277) were also observed in marine organisms from South Korea [4], suggesting the bioaccumulation potential for OH-PBDEs were less than those for MeO-PBDEs. However, the BSAF values of MeO-PBDEs (0.002–0.14) and OH-PBDEs (0.004–0.18) in marine organisms from polar regions were much lower than those calculated in Liaodong Bay and South Korea [3]. The wide range in the estimates for BSAF values for PBDE metabolites in various marine organisms may be the result of species-specific differences in metabolism and selective excretion abilities.

### 5.2. BMF and TMF

Biomagnification factor (BMF) and trophic magnification factor (TMF) are commonly used to investigate the biological magnification behaviour of pollutants in the food chain. BMF was defined as the ratio of the average lipid-normalized concentration between predator and prey [80]. TMF was used to describe the biomagnification and was calculated by the correlations between average lipid-normalized concentration and trophic levels (TLs) [81]. BMF and TMF values >1 indicate biomagnification, whereas values between zero and one imply that the compound is present throughout the food web but is not being biomagnified.

In marine mammals from the southern North Sea, BMFs calculated for harbour porpoises were higher for 6-MeO-BDE 47 (2.2–23.3) than for 2′-MeO-BDE 68 (0.4–5.0), although this was not obvious for harbour seals. Median BMF values were all <1 in harbour seals (0.1–0.3), whereas they were >1 in harbour porpoises (1.1–6.3). BMFs for both MeO-PBDEs were <1 in harbour seals, suggesting the possible breakdown of MeO-PBDEs in harbour seals rather than in harbour porpoises or a higher absorption rate in harbour porpoises [41]. BMFs for OH-PBDEs (1.3 ± 1.0) and MeO-PBDEs (1.0 ± 0.5) from ringed seal blubber to polar bear adipose tissue indicated that OH/MeO-PBDEs bioaccumulated and were not biomagnified from ringed seals to polar bears [59].

Kelly et al. (2008) studied, in the Hudson Bay region of northeastern Canada, MeO-PBDEs by analyzing microalgae, bivalves, Arctic cod, sculpin, salmon and beluga whales. Concentrations of 2′-MeO-BDE 28, 6′-MeO-BDE 49 and 6′-MeO-BDE 66 did not increase significantly with TL whereas 2′-MeO-BDE 68 and 6-MeO-BDE 47 exhibited significant concentration increases over the food web in Hudson Bay, with TMFs equal to 2.3 and 2.6, respectively [8]. TMFs > 1 for MeO-PBDEs were also found in the marine food web from Sydney Harbour in Australia (2.9, 2.4 and 3.3 for ƩMeO-PBDEs, 6-MeO-BDE 47 and 2′-MeO-BDE 68, respectively), the southern part of South Korea (2.36 for ƩMeO-PBDEs), Arctic (2.4, 2.2 and 1.5 for ƩMeO-PBDEs, 6-MeO-BDE 47 and 2′-MeO-BDE 68, respectively) and Antarctica (2.2 and 2.5 for ƩMeO-PBDEs and 6-MeO-BDE 47, respectively) [3,4,36]. However, in Antarctica, concentrations of 2′-MeO-BDE 68 decreased slightly when TL rose over the food web, with a TMF of 0.7 [4].

To our knowledge, TMFs < 1 for OH-PBDEs were found in all the marine food weds reported. For example, in the Liaodong Bay food web, lipid-normalized concentrations of 6-OH-BDE 47 and 2′-OH-BDE 68 decreased significantly with increasing trophic levels. TMF values for the two congeners were 0.21 and 0.15, respectively. The trophic dilution tendency of OH-PBDEs were also found in a seaweed−invertebrate−fish food web in the Arctic (TMF = 0.81 for ƩOH-PBDEs) and in an algae−invertebrate−fish food web in the Antarctic (TMF = 0.56 for ƩOH-PBDEs). The lower TMF values for OH-PBDEs in marine organisms are consistent with previous metabolic analysis results [82], suggesting that the metabolic rate would be a main factor affecting trophic magnification and dilution. However, additional investigations should be conducted on complex food webs with several TLs to further understand the key mechanisms governing the environmental behaviour of OH-PBDEs and MeO-PBDEs.

## 6. Summary and Future Perspectives

OH/MeO-PBDEs were widely ubiquitous in the marine environment. Although research on the source and distribution of these two compounds have been conducted, the source, environment behaviour, toxicity and risk of human exposure are poorly understood.

To better understand and manage the potential environmental risks associated with OH/MeO-PBDEs, the following areas of research should be prioritized. First, the producers of OH/MeO-PBDEs and the synthesis pathway must be further investigated. The present studies have shown that OH/MeO-PBDEs may come from the biosynthesis of algae and sponges or their symbiotic bacteria; however, the main producer and the synthesis pathway is not clear. It is important to investigate the main producer and the synthesis pathway to better understand their environmental behaviour, biotransformation and ecological risk.

It is important to understand the overall fate, including biotransformations, of OH/MeO-PBDEs in biota to assess their potential risk. Unfortunately, the toxicokinetics of OH/MeO-PBDEs are poorly studied. Labelled tracers, such as compound-specific carbon isotopes, are typically used in the study of toxicokinetics [83]. Therefore, the toxicokinetics of OH/MeO-PBDEs using labelled tracers should be considered as an important topic in future research.

Finally, more research will be needed to investigate the toxicology and human exposure risk of OH/MeO-PBDEs. OH/MeO-PBDEs are found in everyday dietary items, including marine fish, mammals and even in fish oils consumed as nutritional supplements. The ecological risk assessment of OH/MeO-PBDEs on the basis of toxicology will be one of the important topics in the future.

## Figures and Tables

**Table 1:** Concentrations of OH/MeO-PBDEs in seawater (pg/L).

Location	Sample Time	N	ƩOH-PBDEs		ƩMeO-PBDEs		Reference
			Range	Mean/Median	Range	Mean/Median	
The Arctic Yellow River Station	2010–2014	3		3.0 ± 1.8		nd ^a^	[3]
Southern coast of South Korea	2015	43	nd—20.2	7.4	nd—8.18	1.03	[23]
Southern part of South Korea	2016	16	nd—18.5		nd—8.18		[4]
San Francisco Bay, USA	2013	25	nd—40				[10]
Great Barrier Reef, Queensland/Australia	2007–2008	16				103 ^b^	[12]

^a^ Not detected. ^b^ Sum of the estimated mean concentrations of 6-MeO-BDE 47, 2′-MeO-BDE 68 and 2′,6-diMeO-BDE 68.

## Data Availability

Not applicable.
